# Editorial: Dog filariosis: the threat walks not only in the blood stream

**DOI:** 10.3389/fvets.2023.1258004

**Published:** 2023-07-21

**Authors:** Ettore Napoli, Gianluca D'Amico, Rafael Antonio Nascimento Ramos

**Affiliations:** ^1^Department Veterinary Sciences, University of Messina, Messina, Italy; ^2^Department of Parasitology and Parasitic Diseases, University of Agricultural Sciences and Veterinary Medicine Cluj-Napoca, Cluj-Napoca, Romania; ^3^Laboratory of Parasitology, Federal University of the Agreste of Pernambuco, Garanhuns, Brazil; ^4^Department of Veterinary Pathobiology, School of Veterinary Medicine and Biomedical Sciences, Texas A&M University, College Station, TX, United States

**Keywords:** *Dirofilaria immitis*, *Dirofilaria repens*, *Acanthocheilonema reconditum*, *Cercopithifilaria* spp., epidemiology, canine heartworm disease

The scientific literature on dog filariasis has expanded considerably in the last decade. From a search on the most used database, Pubmed (https://pubmed.ncbi.nlm.nih.gov/?term=dog+filariosis), 2,179 scientific articles were published between 1902 and 2022, of which 31.8% (*n* = 693) was produced the last decade (2012–2022). The question that arises is: what has led to this increased scientific production of *Canine filariasis*? *Canine filariasis*, or filarial nematode infection in dogs, may be caused by different species, including the most pathogenic *Dirofilaria immitis*. Other filarids characterized by microfilariae in the blood, such as *Dirofilaria repens* and *Acanthocheilonema reconditum*, are just as consequential due to potential zoonosis and species misdiagnosis. However, alluded to in the title of this article collection, “*The threat walks not only in the blood stream*”; not all filarid microfilariae dwell within the blood. Indeed, considerable attention has been drawn to the genera *Onchocerca* and *Cercopithifilaria*, whose microfilariae are skin-dwelling.

It is well-known that all nematodes of the Family Onchocercidae (Spirurida) are transmitted by arthropod vectors (e.g., *Dirofilaria* spp. by mosquitoes and *Cercopithifilaria* spp. by ticks) (1, 2), or there is strong evidence indicative to this type of transmission (e.g., *O. lupi*). From an epidemiological perspective, the vectorial transmission of pathogens has dramatically shifted due to the recent climatic changes that have influenced the distribution of arthropod populations (3). One of the consequences of global warming is the broadening area of colonization of arthropod vectors (e.g., mosquitoes, ticks, fleas), with the invasion of new areas and eventually increase of their vector capacity. For example, this spreading has been clearly observed for *D. immitis* and *D. repens* in Europe (2, 4, 5). In the past, *D. immitis* was considered endemic only in the Mediterranean countries. However, the distribution patterns have recently changed and expanded toward eastern and north-eastern European countries (6–8). Following the same trend, *D. repens* has increased its prevalence in areas where it has already been reported, and its distribution range has expanded into new areas of Europe, with new cases in both dog and human hosts (9).

*Dirofilaria immitis*, the main representant of this genus, has been reported to predominantly infecting dogs, other animal species (e.g., cats, wild canids, badger), and humans (5, 10, 11). In dogs, adult nematodes localize primarily in the pulmonary arteries, causing a severe and debilitating cardio-pulmonary condition known as “heartworm disease” (12). Females release first-stage larvae (microfilariae) in the bloodstream of the vertebrate host. Mosquito vectors (e.g., *Culex, Aedes, Ochlerotatus*, and *Anopheles*) ingest these microfilariae and develop for ~14 days to become infective third-stage larvae (13). The success of larval development within the mosquito host depends on temperature (14). Thus, development ceases if the temperature falls below the minimum threshold (14°C). The total amount of environmental heat required for developing *D. immitis* can be expressed in degree-days above the threshold temperature (Heartworm Developing Units, HDU). In general, the transmission model assumes that an accumulation of 130 HDUs within a maximum period of 30 days (average life span of the mosquito host) is needed for *D. immitis* to reach infectivity (15).

*D. repens* has also been considered a notable mosquito-borne nematode, responsible for canine subcutaneous filariasis in dogs and sporadically in humans (16, 17). Adult nematodes are mainly found in the subcutaneous or intramuscular connective tissue of vertebrate hosts (2); however, the presence of this nematode has been recently reported in unusual anatomical locations such as the pelvic and mesentery cavity, eyes, and testis (18–21). Similar to *D. immitis*, the causative agent of subcutaneous dirofilariosis is transmitted by mosquitoes (e.g., *Aedes* and *Culex*) (22, 23). In several vertebrate hosts, including humans, most inoculated microfilariae die or migrate in host tissues originating from subcutaneous nodules (23, 24). These lesions are characterized by focal edema and erythema triggered by the presence of the parasite (25–28).

Although *Dirofilaria* species have been widely studied, other filarial nematodes are known to parasitize dogs, such as *O. lupi* (29–31). This nematode is an emergent species infecting domestic dogs and has been identified in Europe, the Middle East and North America (32–39). This parasite's infection in canids is characterized by the presence of ocular lesions, including subconjunctival granulomas (33, 40, 41). There are many gaps in the epidemiology of *O. lupi*, mainly because information about its vector is incomplete (42). Until recently, only *Simulium tribulatum* was suggested as the putative vector of this filarial worm in California, United States (US) (43).

Currently, three species of *Cercopithifilaria* have been recognized in dogs: *Cercopithifilaria grassii, Cercopithifilaria bainae*, and *Cercopithifilaria* sp. II (44–46). *C. bainae*, the most studied species within this genus, was first described in a dog from Brazil (45) and, in the last decade, was reported to infect dogs in Italy (46). Current evidence supports a broader distribution of *C. bainae* than previously known (29, 47–49). Unlike most filarioid species, *C. bainae* is vectored by *R. sanguineus* sensu lato (s.l) ticks (47, 50). Although its pathogenicity had not been fully elucidated, parasitism by this nematode has been associated with polyarthritis (51), dermatitis (29, 52), and the presence of a giant cutaneous cyst in dogs (53). The importance of other filarioid genera, such as *Acanthocheilonema* and *Brugia*, must be addressed. Despite low pathogenic relevance, *Acanthocheilonema* spp. may cause misdiagnosis in regions where *Dirofilaria* spp. are also endemic, and *Brugia* spp. has significant zoonotic implications (53).

In this Research Topic, the importance of canine filariasis have been demonstrated in five unique articles. Two literature reviews highlight the importance of cutaneous filarioids in the US (Gruntmeir et al.) and the other focus on *Dirofilaria* in the UK (Panarese et al.). Two research articles related to the risk of infection (Ciuca et al.) and new distribution patterns of *Dirofilaria* species in Italy (Napoli et al.), and finally, the evidence of the putative role of biting midges as vectors of *O. lupi* in the US (Roe et al.). This Research Topic will highlight significant aspects of canine filariasis to serve as a knowledge resource for any remaining gaps in the scientific literature that require attention.

## Author contributions

All authors listed have made a substantial, direct, and intellectual contribution to the work and approved it for publication.
